# Infant With Severe Penoscrotal Hypospadias: A Complex Case of Genital Ambiguity and Mistaken Identity

**DOI:** 10.7759/cureus.15191

**Published:** 2021-05-23

**Authors:** David Nelwan, Conner Mount, Bradly Morganstern, Jacqueline T Chan

**Affiliations:** 1 Urology, Medical College of Georgia at Augusta University, Augusta, USA; 2 Pediatric Endocrinology, Medical College of Georgia at Augusta University, Georgia, USA

**Keywords:** ambiguous genitalia, disorders of sexual development, pediatric urology, pediatric endocrinology, hypospadias

## Abstract

Individuals with an incongruence of their chromosomal sex and genital appearance are classified as having a disorder of sexual development (DSD), and they often present with ambiguous genitalia. The diagnosis and management of DSD patients are usually challenging and require a multidisciplinary approach. Gender assignment should not be based solely on physical exam and imaging but also on the genotype and hormonal function of the gonads. We present an infant born with ambiguous genitalia; the parents were told they were having a male infant during the prenatal ultrasound but at birth, the infant was found to have female-appearing external genitalia with no palpable gonads. MRI of the abdomen was inconclusive, but further workup, including karyotype, hormonal function, and intraoperative evaluation, was consistent with a male infant. He was, therefore, subsequently assigned to the male sex.

## Introduction

Individuals with an incongruence of their chromosomal sex and genital appearance are classified as having a disorder of sexual development (DSD). The incidence is estimated at 1:2500-5000 live births [1-2], with a higher incidence in areas with greater consanguinity [3-4].

Gonadal development starts at fertilization when chromosomal sex is established. It was at first assumed that gonadal differentiation is solely governed by the absence or presence of the sex-determining region Y (SRY) gene located on the Y chromosome, which transforms a bipotential gonad to testes. It is now believed that gonadal determination involves a complex of gene expressions and transcription factors causing the activation of the testicular pathway while at the same time inhibiting the ovarian conduit (or vice versa). As the testes are established, Leydig cells and Sertoli cells are formed and produce testosterone and anti-Mullerian hormone (AMH), respectively, around the eighth week of gestation. This leads to the regression of the paramesonephric ducts and differentiation of the Wolffian duct into male reproductive organs. The absence of AMH and low levels of testosterone leads to the persistence and differentiation of the paramesonephric ducts into fallopian tubes, uterus, and superior portion of the vagina [5]. Given the numerous steps in male differentiation starting with a Y chromosome, a disruption at any stage could result in a DSD with an XY karyotype.

Infants born with DSD usually present with ambiguous genitalia, in which the external genitals cannot be clearly identified as male or female. Once life-threatening disorders have been ruled out, evaluation of an infant with ambiguous genitalia requires a multidisciplinary team to assign an appropriate sex and gender assignment.

We present a case of an infant born with ambiguous genitalia due to severe hypospadias, requiring complex medical investigation and decision-making for proper gender reassignment.

## Case presentation

An infant was born at 34 weeks gestational age via C-section secondary to preeclampsia with oligohydramnios. The patient was noted to have intrauterine growth restriction and was small for gestational age. At the 17-week prenatal ultrasound, parents were told they were having a male infant. At birth, however, external genitalia appeared female with no palpable gonads. The newborn screen was normal. An ultrasound was performed and noted a uterine-appearing structure and a possible right ovary. Parents were thus advised to name and raise the patient as a female at discharge from the neonatal intensive care unit. However, the karyotype later came back 46, XY.

The patient was seen by pediatric endocrinology at four weeks of age. At this time, the infant was starting to gain some weight. The genital exam was similar to that at the time of birth but with marked clitoromegaly. Because the initial ultrasound showed a uterine structure and possible ovaries with no mention of male structures on an XY infant, the SRY gene was sent and came back positive. The infant had a follow-up at eight weeks of age, at which time, the exam showed a more phallic structure. The hormonal workup revealed normal testosterone and AMH levels of 82.42 ng/dL and 96 ng/mL, respectively, with an undetectable estradiol level, consistent with an infant with intact testicular function. LH and FSH were also normal at 2.56 mIU/mL and 4.26 mIU/mL, respectively. An MRI of the abdomen and pelvis and was read as (1) Gonadal tissue within the bilateral inguinal canals with mixed testicular and ovarian MR characteristics; (2) Clitoromegaly; (3) Soft tissue structure interposed between the rectum and bladder likely representing the vagina; (4) No uterus or abdominopelvic gonadal tissue identified.

**Figure 1:**
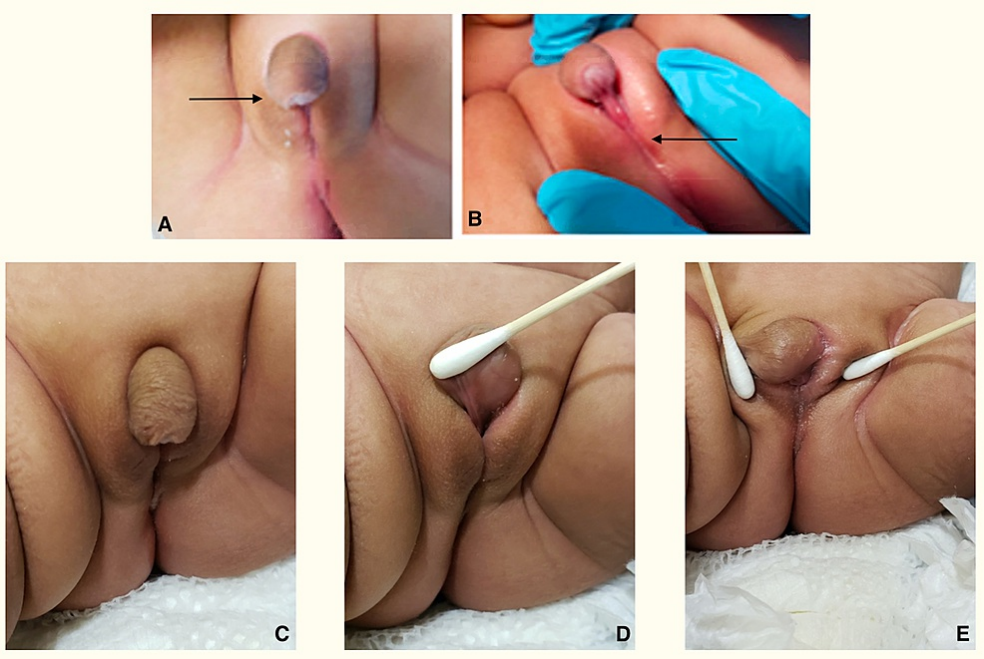
Physical exam (A) At four months, severe penoscrotal hypospadias with an underdeveloped bifid scrotum, ~90-degree chordee, penile transposition, dorsal hood, and non-palpable testicles; (B) Atretic urethral plate and penoscrotal meatus without introitus or evidence of vagina; (C-E) At five months, a notable increase in phallus length

The patient was subsequently referred to pediatric urology and seen at five months of age. The physical exam noted severe penoscrotal hypospadias with bifid scrotum (Figure 1A); incomplete penoscrotal transposition (Figure 1B); bilateral undescended testicles high in the inguinal canal; and the “clitoromegaly” on MRI appeared to actually be a phallic structure with chordee; what appeared to be a vagina on prior exams and imaging was favored to be a utricle. Following extensive discussion with the family about the options available to them, their stated goals were to get definitive diagnostic testing to determine the sex of their child, and the patient was scheduled for diagnostic laparoscopy and gonadal biopsy. Preoperative cystoscopy noted: (1) a verumontanum without an identifiable utricle; (2) bilateral ureteral orifices in orthotopic position; (3) no gross bladder abnormalities. Intraoperative findings included: (1) bilateral spermatic vessels and vas deferens entering open rings; (2) no uterus, fallopian tubes, ovaries, or any other Müllerian structures identified; (3) normal-appearing right testicle and epididymis with tubules noted upon small testicular incision. The right testicle was then biopsied and orchiopexied, and biopsy results confirmed testicular tissue. Given the presence of a Y chromosome, functioning SRY gene, hormonal levels, reproductive anatomy on surgical exploration, and confirmatory gonadal biopsy, it was justifiable for the family to assign their child to the male sex and raise him as such. The patient underwent stage 1 hypospadias repair at 10 months of age, including chordee repair with Byars and Dartos flaps, AlloDerm and EpiFix graft placements, as well as complex scrotoplasty.

## Discussion

The diagnosis and management of DSD patients can often be challenging and requires a multidisciplinary approach, usually including an endocrinologist, urologist, and psychologist.

DSD evaluation is warranted for grossly atypical genitalia, but there are less conspicuous presentations for which it is also recommended. With predominantly male genitalia, further investigation is indicated for bilateral undescended testes, severe hypospadias, or hypospadias with undescended testes and/or micropenis. With predominantly female genitalia, further evaluation should be done for significant clitoromegaly, posterior labial fusion, or palpable gonads inguinally or in the labioscrotal folds.

Infants born with ambiguous genitalia are most commonly categorized as either undervirilized males or virilized females. Therefore, careful physical examination and family history of consanguinity or prenatal androgen exposure is important. It is also important to get a rapid karyotype and/or SRY gene probe, allowing for categorization as XX DSD, XY DSD, or sex chromosome DSD [4].

Initial testing should be to rule out congenital adrenal hyperplasia (CAH) due to the potential for a life-threatening adrenal crisis. This includes a 17-hydroxyprogesterone level (17-OHP) to test for the most common form of CAH (21-hydroxylase deficiency), as well as electrolytes to check for hyponatremia and hyperkalemia. Female infants usually present with virilization and hence are typically diagnosed early, whereas male infants are usually missed and are either seen at five to 10 days of life with an adrenal crisis or when the newborn screen comes back abnormal. Our patient was initially thought to be a virilized female due to what appeared to be an enlarged clitoris, absence of inguinal structures or testes, and the initial US showing a uterus and an ovary. The newborn screen came back normal, as did 17-OHP.

Gonadal activation is ideally investigated when testosterone peaks at one to three months of life, also referred to as mini-puberty. Gonadal function is investigated via levels of luteinizing hormone (LH), follicle-stimulating hormone (FSH), testosterone, dihydrotestosterone (DHT), and AMH. LH is a good indicator of the hypothalamic-pituitary-gonadal axis (HPG); low levels of LH could indicate hypogonadotropic hypogonadism while elevated LH levels represent inadequate androgen activity/synthesis such as in complete androgen insensitivity syndrome (CAIS)/partial androgen insensitivity syndrome (PAIS) or gonadal dysgenesis. The serum AMH level is valuable for assessing gonadal function and helps explicate the level of defect in an XY DSD patient. A low level of AMH in an XY DSD suggests gonadal dysgenesis [6-7].

First-line imaging is an ultrasound to evaluate the presence or absence of a uterus, as well as to attempt the visualization of gonads. However, ultrasound results are user-dependent and can be misleading, as seen in this case, and thus an MRI of the abdomen/pelvis is typically more reliable. It is therefore important to emphasize that gender assignment should not be based solely on imaging but also on the genotype and hormonal function of the gonads. In this case, the initial ultrasound stated the presence of a uterus. The differential for an XY patient that does have a uterus would include XY gonadal dysgenesis or Swyer syndrome, persistent Müllerian duct syndrome, and ovotesticular DSD. The failure of the primitive gonad to differentiate into testes in XY gonadal dysgenesis would result in decreased or absent AMH production and thus the development of a uterus and other paramesonephric structures. The most common etiologies are mutations in SRY, NR5A1, and WT1 [8]. Patients would also have similar clinical presentations if they had persistent Mullerian duct syndrome due to a mutation in the AMH gene or receptor [9-10]. With an incidence of less than 1:20000, the ovotesticular subtype is among the rarest DSD, particularly with an XY karyotype. The cause is often unclear, with some cases reporting mutations in SRY, MAP3K1, and DMRT1 [9]. In complicated cases, such as this one, and especially with equivocal imaging, direct visualization of internal structures via laparoscopy/laparotomy is of most diagnostic benefit, often with gonadal biopsy.

After presenting initially with ambiguous genitalia, our patient’s physical exam at two months of age appeared more virilized, likely due to the surge of male hormones or mini-puberty. A complete hormonal and genetic workup revealed an XY infant with intact testicular function. It was more evident at that time that the infant had severe penoscrotal hypospadias. Hypospadias is a congenital malformation in males where the urethra does not open at its usual position at the tip of the glans, instead found anywhere in the ventral midline as far back as the perineum. In severe cases of hypospadias, especially with a concomitant penoscrotal transposition, the atypical anatomy may be mistaken for clitoromegaly with a vestibular groove and interpreted as female genitalia.

There are several non-medical aspects of the diagnosis and management of DSD patients that may be overlooked but are of particular importance. Setting expectations for the timeframe of an extended workup, as well as awareness and acknowledgment of the stress of a DSD workup and diagnosis, are key starting points for family involvement in the process [10]. It is also helpful to refrain from technical explanations while still providing basic information about normal sexual development in utero and underscoring the fact that there is a biologically understandable etiology of the clinical presentation [11].

It is recommended that families delay typical rites like birth announcement and completion of the birth certificate until a diagnosis is finalized. Giving a gender-neutral name is usually advised as well. Parental access to counseling and psychosocial support is essential to help families handle the unique situation of raising an infant with DSD [4]. Decisions about sex in rearing and surgical considerations are perhaps the most challenging aspects of the management of a child with a DSD. Of particular difficulty is the fact that these decisions often take place in early infancy or childhood, but gender identity may not crystallize until adolescence or later. While gender identity can be relatively predictable in certain DSDs like 46, XX CAH, that is not always the case, and it is also complicated by factors like prenatal androgen exposure, reproductive and sexual capability, and sociocultural considerations [12-13]. Surgical decision-making should follow extensive specialized counseling, with some DSD advocacy groups contending that surgery should take place only after the child has formed a gender identity [14].

## Conclusions

Infants born with ambiguous genitalia require a multidisciplinary approach. Proper evaluation includes a thorough physical exam, karyotype, hormonal workup, imaging, and in complex situations such as this case, surgical exploration. Parental counseling is key to helping families cope with the situation and provides the support they need to make informed decisions about their child's future.
